# The Complex Influences on How We Care for Farmed Fish

**DOI:** 10.3389/fvets.2021.765797

**Published:** 2022-01-04

**Authors:** James F. Turnbull

**Affiliations:** Faculty of Natural Sciences, Institute of Aquaculture, University of Stirling, Stirling, United Kingdom

**Keywords:** fish, welfare, salmon, behavioral theory, aquaculture, behavioral science

## Abstract

As a veterinarian and academic in aquaculture, in my personal experience, most farmers are concerned for their animals and want to take good care of them. There has been substantial improvement in the welfare of farmed fish in recent decades, but improvements have been inconsistent across culture systems and species. Where there has been a lack of progress, it is not simply due to the more obvious barriers, for example, lack of clear messages, lack of effective dissemination, or cost of implementation. Why have the good intentions of farmers and research by academics failed to improve the care of many farmed fish? The reasons would appear to be complex; however, human behavioral theory (this term is used to differentiate from animal ethology) offers both a conceptual framework and practical guidelines for improving the care of fish by influencing the behavior of farmers. Here, I present some background context and apply human behavioral theory to examples of on-farm care of fish.

## Personal Perspective On Fish Welfare and How To Protect It

I have been fortunate to work as a veterinarian, researcher, and teacher in aquaculture in many parts of the world for over three and a half decades. In all that time, meeting and talking with many fish (and shrimp) farmers, I have never encountered one who intentionally mistreated their animals. Most expressed considerable concern for their animals [e.g., (1)]. Here, I refer to farmers as the people actively involved in the day-to-day care of the animals. While many owners or investors also share this concern for the animals, in some cases, these individuals or organizations have a more abstract relationship with the animals and have priorities driven by business concerns.

While there has been some significant progress in the practical care of farmed fish over the last three decades, progress has been inconsistent across culture systems and species. I have observed many cases of extremely poor fish welfare, especially around the time of harvest and slaughter. In my experience, such treatment of fish did not appear to be due to malice; in some cases, it was due to lack of understanding or resources, but in many cases, it was failure to implement existing viable strategies. Even in the best farming systems, there is still the potential for improvements in fish welfare and the question remains how should we achieve such gains?

Personally, working in both the applied and academic contexts, the relationship between improved academic understanding of welfare and practical care has been far from clear to me. Where translation from academic to applied context has been ineffective, it was not always due to the more obvious barriers, for example, lack of clear messages, lack of effective dissemination, or cost of implementation. While complex and unresolved scientific or ethical arguments are difficult to translate into husbandry practices, not all aspects of animal welfare are complex or still widely debated. Even where scientific issues are clear and effectively disseminated, and there is a demonstrable benefit for the business, progress remains limited.

Therefore, there must be other barriers to the translation from academic endeavors to practical animal welfare. Reflecting on how theoretical understanding has influenced my own practical actions regarding fish welfare, I find that some information has remained academic theory while other examples have affected my behavior. For example, work by Victoria Braithwaite on simple manipulations in the environment and feeding of juvenile cod (*Gadus morhua*) (2) opened my eyes to the possibility that fish might be capable of more complex and rewarding lives than I had previously thought possible, but this did not significantly affect my actions. In contrast, discussions on pain in fish based on studies conceived by Victoria Braithwaite and Mike Gentles (3, 4) convinced me that fish should be given the benefit of the doubt with regard to pain. As a result, I have changed my behavior, with the prevention, or alleviation of (potential) pain in fish becoming a personal priority.

Seeking to understand why some evidence affects behavior and some does not (personally and at an industry or national level), I looked outside the field of animal health, welfare, and husbandry for a conceptual framework. The relationship between information and subsequent action by people is central to public policy and many other areas. Further reading on influencing behavior introduced me to what is referred to as “behavioural theory” in the literature. Here, I use the term “human behavioural theory” to differentiate it from animal ethology. There is a large body of literature and many examples of successful application of the theory to influence peoples' behavior.

Human behavioral theory is used to understand and influence human behavior by governments and others. It demonstrates that evidence is only one aspect of the suite of influences and contexts that affect our decisions. The concept that our decision making can be influenced was developed by Richard Thaler, a Nobel Prize-winning behavioral economist. The concept is based on influencing our behavior by utilizing our cognitive shortcuts and biases. Influencing people's behavior is a complex issue and has the potential to be used for unsavory purposes; however, given its success and positive benefits in many areas, it also has the potential to improve the way people care for fish and other animals.

Below, I present evidence for some of the statements above (e.g., farmers want to take care of their fish, barriers to applying better care, and example of a successful welfare initiative) and then apply human behavioral theory to examples of applied fish welfare. The aim is to explore the potential of this approach with a view to stimulating further work in this area and facilitate additional gains in the welfare of farmed fish.

## Barriers To The Implementation of Improved Care of Farmed Fish

### Do Farmers Want to Take Care of Their Fish?

Our caring relationship with animals appears to extend back tens of thousands of years, as discussed by Bradshaw (5) and providing good animal welfare is important for people who have an affinity with animals. Attitudes to care for animals, whether pets or livestock, is not ubiquitous across cultures or individuals, and even in individuals, attitudes may change over time. While farmers are often more concerned with health and productivity rather than less easily measured aspects of welfare, participation in welfare schemes can increase awareness and concern for ethical and moral aspects of welfare (6). In terrestrial animal farming, it has been demonstrated that believing the animals under one's care are intelligent and can benefit from positive experiences will lead to a more pleasant experience for the farmer and more positive behavior toward the animals (7). Conversely, working in an environment where the animals are treated as economic units or purely mechanical devices can lead to a deterioration in the relationship between the farmer and the animals (7). This emphasizes that information or even understanding are not the only determinants of our behavior toward animals.

### Is the Lack of Academic Consensus a Barrier to Improved Practice on Farms?

Several key concepts that are important when conceptualizing and protecting fish welfare are still debated in the academic literature. There is a lack of agreement on definitions for, or the existence of sentience, consciousness, cognition, pain, positive, and negative emotions in fish. Therefore, the debate is likely to continue for some time (8). In the absence of consensus, there may be no clear path to follow, and disagreements may throw doubt on, or disguise related but more widely agreed aspects of welfare. However, the public's attitudes to animal welfare are not always rational or based on scientific evidence or theories (9) and there is broad agreement on some key welfare issues.

Humane slaughter is widely accepted as essentially for good animal welfare (10) and potentially achievable but is still not universally adopted. It is still limited to a very small proportion of the fish killed for human consumption every year. This is an issue that affects a very large number of animals; estimates are highly uncertain, but may be in the tens or hundreds of billions (11). The complex relationship between the evidence base and our behavior toward animals would suggest progress is not entirely dependent on academic consensus.

### Is Lack of Effective Dissemination a Barrier to Improved Practice on Farms?

There are examples of effective information dissemination between academics and industry. These range from original research papers, through summaries of the information (12), and assurance schemes with detailed sets of standards for farming practices (13), to practical applied training (14), although such training is still limited to a small number of options. However, these are limited in terms of husbandry systems and geography. There is, for example, widespread adoption of higher welfare standards by the Atlantic farming industry in the northern hemisphere and evidence of growing awareness in some tilapia faming sectors but a lack of any significant progress in much of Asian aquaculture. It would therefore seem that lack of dissemination may be a barrier to progress in some contexts.

### Is the Cost of Implementation a Barrier to Improved Practice on Farms?

Improving fish welfare can increase productivity and allow access to markets and therefore does not always have a net cost. Even when there is a net cost, there may be good reasons for bearing that cost, including compliance with legislation and worker satisfaction.

There is a common intuitive notion that animals that are well-cared for will be more productive and there is also quantifiable evidence for the relationship (15, 16). However, there are still few examples where bioeconomic models have been used to clearly identify the costs and benefits of improving farmed fish welfare (17). There are additional business benefits, including fulfilling the demands from the value chain. Demonstrating that the fish are being provided with adequate care has a value in terms of both protecting and developing markets (15, 18). As a result, businesses have seen advantages in participation in accreditation or certification schemes that allow them to market fish with a high standard of welfare, for example, RSPCA Assured [e.g., (13)] and the Code of Good practice for Scottish Finfish Aquaculture (19).

Legislative instruments are another incentive for protecting the fish welfare even if there is a net cost. Regulation has been introduced to protect the welfare of farmed animals, for example, in Europe [e.g., (20–22)] backed up to some extent by national legislation [e.g., UK (23, 24).

As discussed above, farmers may wish to take care of animals based on personal beliefs, and therefore, the benefits of better fish welfare may go beyond improved productivity or profits. For example, “*happy fish equals happy farmer, and a happy farmer equals job satisfaction/employee retention*” (J Wiper. Cooke Aquaculture. Personal communications).

### What Has Worked?

Despite the uncertain links between information and impact, some initiatives would appear to have been successful at disseminating information and promoting better welfare practices. The UK RSPCA Assured program (formerly Freedom Foods) has been adopted by more than 70% of the Scottish salmon farming industry (25) and the standards have also been used as the basis for other international initiatives including Fishwell (12) and MERCK animal health's Aqua Care 365 program (14). Objectively measuring the impact of the RSPCA scheme is very challenging, given the complex interacting influences involved, but some research is currently examining this problem (26). The scheme is based on comprehensive welfare-based standards that were developed in consultation with a wide range of stakeholders. According to news reports, the level of adoption was, at least in part, driven by the demands of the retail sector. It also provides incentives; following regular inspections and investigations of any complaint, there is the capacity to impose sanctions or remove accreditation. It has a very large formative component where the RSPCA Assured staff work with farmers to help them understand fish welfare and improve the welfare of the fish on their farms. However, it is difficult to determine the value of these various aspects of the scheme without a conceptual framework to understand how they might affect people's behavior.

## Human Behavioral Theory and Farmed Fish Welfare

Human behavior theory offers such a theoretical framework and practical guidelines to better understand the influences on how people care for farmed fish and help us to achieve more effective change in the future. A document on the relationship between public policy and changes in human behavior based in behavioral theory (27) provides a framework (checklist) to examine what affects decisions. The document presents the most robust (non-coercive) influences on our behavior as a mnemonic (MINDSPACE):

**Messenger**—we are heavily influenced by who communicates information.

**Incentives**—our responses to incentives are shaped by predictable mental shortcuts such as avoiding losses.

**Norms**—we are strongly influenced by what others do.

**Defaults**—we “go with the flow” of pre-set options.

**Salience**—our attention is drawn to what is novel and seems relevant to us.

**Priming**—our acts are often influenced by sub-conscious cues.

**Affect**—our emotional associations can powerfully shape our actions.

**Commitments**—we seek to be consistent with our public promises and reciprocate acts.

**Ego**—we act in ways that make us feel better about ourselves.

This is not an exhaustive list, reflective (conscious) and automatic (unconscious) thought processes are affected by different sub-sets of these influences (27).

Human behavioral theory has been used in a wide variety of contexts, for example, reducing the spread of HIV in sub-Saharan Africa. The UK's Department for International Development recognized and utilized the complex drivers of human behavior in a successful scheme to reverse the spread of HIV (28). Another example is the reduction of gang violence in Western Central Scotland. Traditional approaches, such as increased foot patrols and stricter enforcement of knife crime legislation, had a positive effect but it was of limited duration. The Violence Reduction Unit successfully adopted an approach based on a model from the USA using norms and messengers to influence behavior (29).

In the context of MINDSPACE, hypotheses regarding potential influences can be developed and tested to improve strategies for change. Below are some conjectures on why some farmers take better care of their fish or in some cases fail to do so.

### Why Might Some Farmers Take Better Care of Their Fish?

**Messenger**—peers or other respected figures promote good fish welfare.

**Incentives**—good behavior is rewarded, and bad behavior is discouraged.

**Norms**—positive attitudes are emphasized, negative attitudes are played down, promoting a culture of care, for fish and people.

**Defaults**—training in good practices and making it harder to do the wrong thing through appropriate infrastructure.

**Salience**—welfare information is presented in a relevant and interesting way.

**Priming**—a supportive stimulating work environment.

**Affect**—innate affinity with the fish and positive emotional associations among staff.

**Commitments**—making explicit commitments to good fish welfare.

**Ego**—caring for the fish makes them feel good (see Affect).

### Why Might Some Farmers Fail to Take Care of Their Fish?

**Messenger**—lack of peer support and either no promotion of good welfare or promotion by people who are not respected.

**Incentives**—no rewards or punishments for good or bad behavior.

**Norms**—a culture where no one appears to care for the fish or people.

**Defaults**—in the absence of good training and poor infrastructure, the go-to option will probably be the easiest option.

**Salience**—any fish welfare information presented in a dry or irrelevant format.

**Priming**—poor social and physical working environment.

**Affect**—employing people without an innate affinity with fish and either neutral or negative emotional associations.

**Commitments**—lack of any agreement on the need for good fish welfare and lack of appreciation of the consequences for others resulting from bad behavior.

**Ego**—lack of awareness of what is the “right thing” to do.

### Specific Example

We can also look at specific initiatives in this context, for example, the UK RSPCA Assured scheme (formerly Freedom Foods).

**Messenger**—the individuals involved in management and implementation of the scheme have had an extremely good reputation and relationship with farmers.

**Incentives**—the level of adoption (>70% of the industry) was at least in part driven by the demands of the retail sector. The scheme conducts regular inspections and investigations of any complaint and has the capacity to impose sanctions or remove accreditation.

**Norms**—most of the industry is in the scheme and staff training and behavior are components of the assessment.

**Defaults**—good practices have become embedded in standard practices.

**Salience**—these comprehensive but welfare-based standards were developed in consultation with a wide range of stakeholders and are regularly reviewed to keep them relevant.

**Priming** and **Affect**—the scheme has a very large formative component where the RSPCA Assured staff work with farmers to help them understand fish welfare and improve the welfare of the fish on their farms.

**Commitments**—membership of the scheme involves explicit commitment to the standards.

**Ego**—the scheme helps workers to understand what the “right thing” means in terms of fish welfare.

## Conclusion

Working both in the academic and applied arenas of fish welfare gave me a personal perspective on the barriers or challenges to effective communication and implementation. Considering the implications of academic studies through the lens of human behavioral theory has the potential to develop more realistic pathways to impact. In the future, bringing expertise in human behavioral theory together with those interested in applied animal welfare has the potential to improve understanding and develop more effective strategies for change. Whether change is industry wide or more localized, the application of human behavioral theory offers a more effective approach than simply provision of information, training, and incentives.

## Data Availability Statement

The original contributions presented in the study are included in the article/supplementary material, further inquiries can be directed to the corresponding author/s.

## Author Contributions

The author confirms being the sole contributor of this work and has approved it for publication.

## Conflict of Interest

The author declares that the research was conducted in the absence of any commercial or financial relationships that could be construed as a potential conflict of interest.

## Publisher's Note

All claims expressed in this article are solely those of the authors and do not necessarily represent those of their affiliated organizations, or those of the publisher, the editors and the reviewers. Any product that may be evaluated in this article, or claim that may be made by its manufacturer, is not guaranteed or endorsed by the publisher.
